# Multiple Papillomas of the Breast: A Review of Current Evidence and Challenges

**DOI:** 10.3390/jimaging8070198

**Published:** 2022-07-13

**Authors:** Rossella Rella, Giovanna Romanucci, Damiano Arciuolo, Assunta Scaldaferri, Enida Bufi, Sebastiano Croce, Andrea Caulo, Oscar Tommasini

**Affiliations:** 1 UOC Diagnostica per Immagini, Ospedale G.B. Grassi, Via Gian Carlo Passeroni, 28, 00122 Rome, Italy; sebastiano.croce@aslroma3.it (S.C.); andrea.caulo@aslroma3.it (A.C.); oscar.tommasini@aslroma3.it (O.T.); 2 UOSD Breast Unit ULSS9, Ospedale di Marzana, Piazzale Lambranzi, 1, 37142 Verona, Italy; giovanna.romanucci@aulss9.veneto.it; 3 Fondazione Policlinico Universitario A. Gemelli, IRCCS, Dipartimento di Patologia Umana, Università Cattolica del Sacro Cuore, 00168 Roma, Italy; damianoarciuolo@yahoo.it; 4 Seno Clinic, Unità di Senologia Casa di Cura privata Villa Mafalda, Via Monte delle Gioie, 5, 00199 Roma, Italy; assuntascaldaferri@yahoo.it; 5 UOC di Diagnostica per Immagini ed Interventistica Generale, Dipartimento di Diagnostica per Immagini, Radioterapia Oncologica ed Ematologia, Fondazione Policlinico Universitario A. Gemelli IRCCS, Largo A. Gemelli, 8, 00168 Roma, Italy; reagandus@alice.it

**Keywords:** papillary lesions, intraductal papilloma, papillomatosis, underestimation, diagnosis, management

## Abstract

Objectives: To conduct a review of evidence about papillomatosis/multiple papillomas (MP), its clinical and imaging presentation, the association between MP and malignancy and the management strategies that follow. Methods: A computerized literature search using PubMed and Google Scholar was performed up to January 2021 with the following search strategy: “papilloma” OR “intraductal papilloma” OR “intraductal papillary neoplasms” OR “papillomatosis” OR “papillary lesion” AND “breast”. Two authors independently conducted a search, screening and extraction of data from the eligible studies. Results: Of the 1881 articles identified, 29 articles met the inclusion criteria. The most common breast imaging methods (mammography, ultrasound) showed few specific signs of MP, and evidence about magnetic resonance imaging were weak. Regarding the association between MP and malignancy, the risk of underestimation to biopsy methods and the frequent coexistence of MP and other high-risk lesions needs to be taken into consideration. Results about the risk of developing breast carcinoma of patients affected by MP were inconsistent. Conclusions: MP is a challenge for all breast specialists, and familiarity with its features is required to make the correct diagnosis. Further studies are needed to evaluate the factors to take into account to plan management, time of follow-up and imaging methods.

## 1. Introduction

Papillomatosis is a rare entity falling into the spectrum of papillary lesions of the breast, and it is defined as the presence of multiple intraductal papillomas (MP). Pathologically, a papilloma is a mass-like projection consisting of papillary fronds attached to the inner of a mammary duct wall by a fibrovascular core covered by ductal, epithelial, and myoepithelial cells [1]. From a morphological point of view, MP share histological features of solitary papillomas, but in contrast to the latter, which usually involve larger ducts, they occur in distal duct-lobular units (TDLUs) [2].

Papillomatosis tends to occur in younger patients than solitary papilloma and accounts for approximately 10% of cases of intraductal papillomas, so only little evidence is available about its clinical and imaging presentation [3].

Several studies reported the association between papillomatosis and the increased lifetime risk of breast cancer, although the reason behind this association is still unclear. Its malignant potential has an impact on its management with uncertainty about the appropriate surgical strategy and follow-up methodology [4].

Therefore, the aim of this review was to provide a summary of the latest evidence about multiple papillomatosis. We focused on its clinical and radiological features, its association with cancer development and its multidisciplinary management.

## 2. Materials and Methods

For the present review, a computerized literature search using PubMed (http://www.pubmed.org, accessed on 31 January 2022) and Cochrane library (http://www.thecochranelibrary.com, accessed on 31 January 2022) was performed up to January 2022. A manual revision of the reference lists was also performed to integrate the initial search with additional studies.

The search strategy included various combinations of the following terms: “papilloma” OR “intraductal papilloma” OR “intraductal papillary neoplasms” OR “papillomatosis” OR “papillary lesion” AND “breast”.

Only articles in English and on human subjects were included.

Excluded were (1) review articles, case reports or case series, replies to study authors, studies published only in abstract form; (2) duplicate publications; and (3) data on non-human subjects. No publication date restriction was used.

Titles and abstracts of search results were examined. When considered suitable, the full text was reviewed. The reference section of retrieved studies was examined to identify additional papers.

Moreover, since the purpose of this systematic review was multiple papillomas or papillomatosis, studies that do not distinguish between MP/papillomatosis and other conditions (such as epitheliosis/juvenile papillomatosis) were excluded.

Two authors (RR and GR) independently conducted the search, screening, quality assessment, and extraction of data from the eligible studies. Disagreements arising during each phase of the study selection or during the quality assessment of the studies were resolved in consensus. If consensus could not be reached, a clinical expert (EB, with more that 15 years of experience in breast imaging) was asked to resolve any disagreements. All necessary data from each eligible study were recorded: demographic data, study design, definition of MP/papillomatosis, clinical findings, imaging findings, upgrade rate to malignancy and outcome (MP recurrence or breast cancer development).

## 3. Results

**Figure 1:**
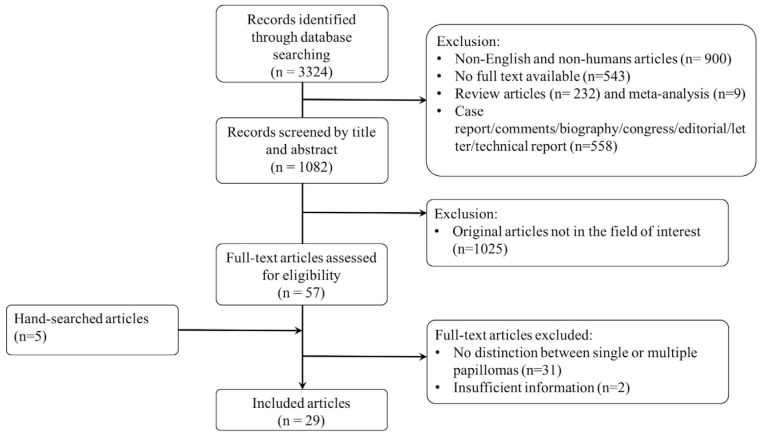
Flowchart of the selection of studies.

Figure 1 shows the flowchart of the selection of studies.

The initial database search of English articles on human subjects identified 1881 articles. A total of 57 full-text articles were assessed after the removal of review articles (*n* = 232) and meta-analysis (*n* = 9) or case report/comments/technical reports (*n* = 502), letters/editorials (*n* = 51), biography/congress (*n* = 5) and original articles not in the field of interest on the basis of title and abstract (*n* = 1025).

From the 57 full-text articles, two articles were excluded for insufficient information (imaging findings and/or patients’ outcomes not reported), 31 studies were excluded because no distinction between single and MP/papillomatosis was made, and one was excluded because MP in the same patient were described as multiple single lesions rather than as a separate entity. After hand searching of references, six additional papers were identified and included.

A total of 29 articles were included in the review. Table 1 shows the characteristics and the main findings of the included studies. 

### 3.1. Clinical and Imaging Findings

MP could manifest as a palpable mass [5,13] or remain asymptomatic [9,23]. Nipple discharge is not so frequent as in single papilloma, occurring in 16–44.4% of cases versus more than 60% in patients with single papilloma (Table 1).

The most common breast imaging methods show few specific signs of MP, and there is little evidence about sensitivity and specificity of different imaging methods for the diagnosis of papillomatosis.

**Figure 2:**
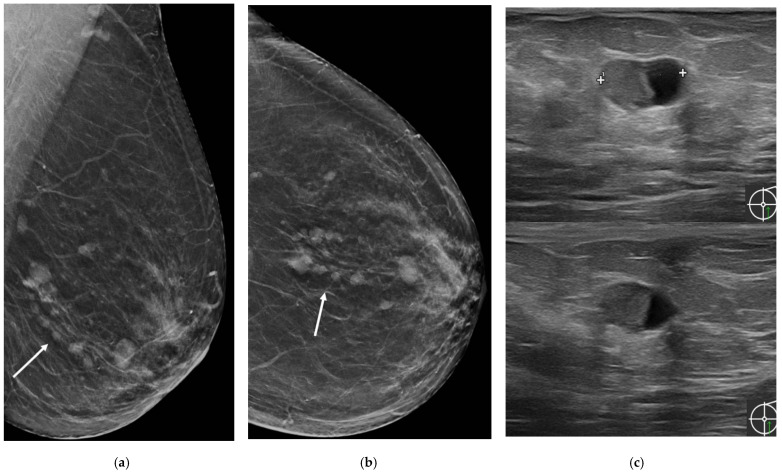
A 55-year-old asymptomatic woman. (**a**) Medio-lateral view and (**b**) cranio-caudal view of screening mammography demonstrated small round or oval opacities in the lower-outer quadrant of the left breast (arrows); (**c**) Ultrasound examination showed multiple intracystic masses.

**Figure 3:**
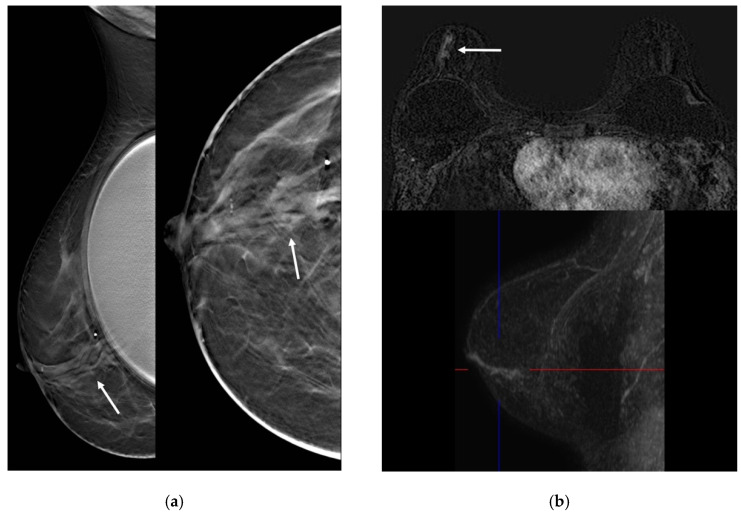
A 56-year-old woman with breast implants presenting with unilateral bloody nipple discharge. (**a**) Tomosynthesis slices in medio-lateral and cranio-caudal view with implant displaced showed multiple dilated ducts in the retroareolar region (arrows). (**b**) MRI showed ductal contrast enhancement (arrow).

Mammography is not a sensitive or specific tool for MP diagnosis; indeed, it could show multiple round- or oval-shaped well-circumscribed opacities, focal asymmetries and clusters of calcifications could be present, but on the other hand, mammography could be silent [23] (Figure 2 and Figure 3).

**Figure 4:**
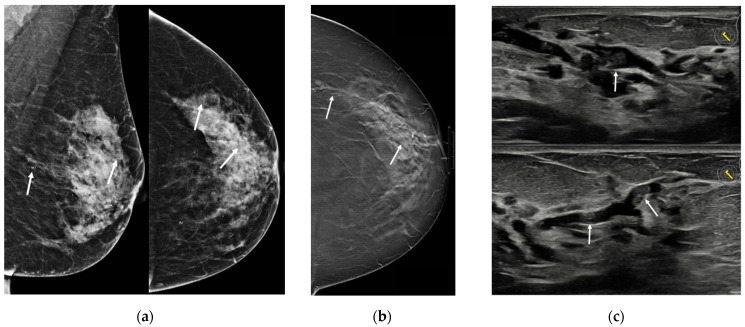
A 55-year-old woman presenting with unilateral bloody nipple discharge. (**a**) Mammography in medio-lateral view and cranio-caudal view showed multiple scattered calcifications (arrows). (**b**) Tomosynthesis slice in the CC view showed multiple dilated ducts from the retroareolar region to the outer quadrants (arrows). (**c**) Ultrasound demonstrated multiple dilated ducts partially filled with intraluminal content (arrows).

At US, the most typical appearance is the evidence of multiple hypoechoic masses with circumscribed margins with ductal relation or intracystic masses or multiple dilated ducts partially or completely filled with intraluminal content; sometimes, a heterogeneous tubular nonmass-like hypoechoic area may be present [26]. The fibrovascular stalk of papillomas can be further assessed with color or power Doppler ultrasound [26] (Figure 4).

There are still no mammographic or ultrasound-specific features to suggest malignant MP transformation.

**Figure 5:**
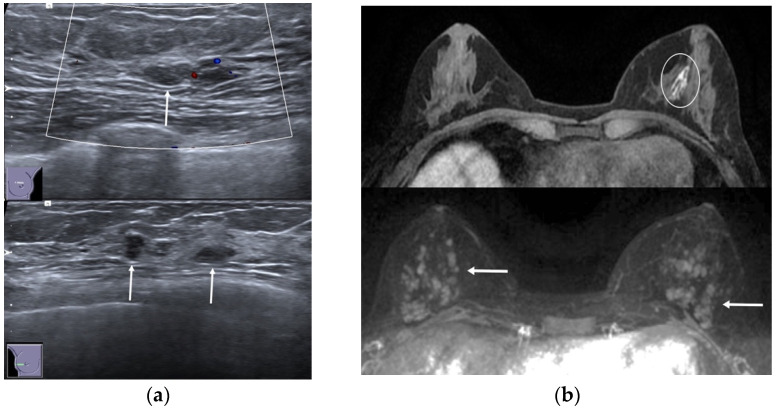
A 36-year-old woman with left bloody nipple discharge. (**a**) Ultrasound demonstrated bilateral multiple hypoechoic masses with circumscribed margins with ductal relation (arrows). (**b**) MRI showed dilated ducts with high T1 signal on pre-contrast sequences (circle) and multiple enhancing masses related with ductal contrast enhancement on post-contrast images (arrows).

**Figure 6:**
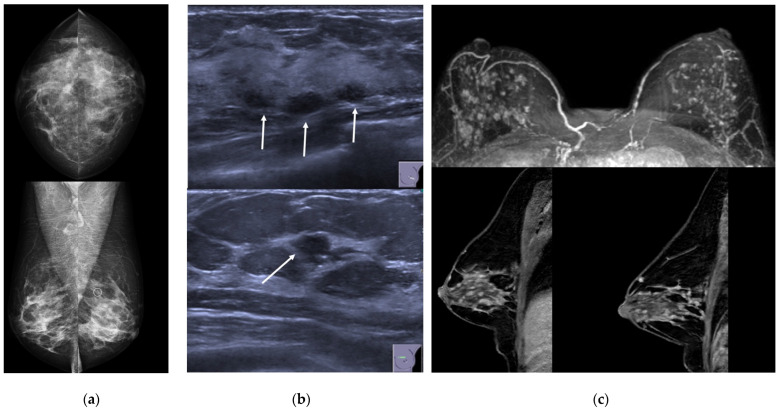
A 43-year-old woman presenting with unilateral bloody nipple discharge. (**a**) Mammography in cranio-caudal and medio-lateral views did not show any mass or asymmetric density, but only a millimetric cluster on calcification in the upper-outer quadrant of the left breast (circle). (**b**) Ultrasound demonstrated multiple small masses with ductal relation (arrows). (**c**) MRI showed multiple enhancing masses associated to ductal contrast enhancement with a “string of pearls” appearance.

Only little evidence about the use of magnetic resonance imaging (MRI) in the diagnosis or evaluation of MP are present in the literature as well as its MRI features. Sarica et al. [26] evaluated the MRI findings of patients with a pathologic diagnosis of papillary lesion, of which there are 11 cases of papillomatosis. The authors reported the following MRI findings of MP: on pre-contrast sequences, dilated ducts (with or without pre-contrast high T1 signal) with an intraductal focal mass on T2 or a mass with crescentic peripheral fluid could be observed; on post-contrast images, a mass related with dilated duct-ductal contrast enhancement or a linear-ductal or segmental contrast enhancement were reported (Figure 5 and Figure 6).

They also found that segmental contrast enhancement was statistically significantly more common in papillary breast carcinoma and papillomatosis groups compared with the papilloma group (*p* < 0.05), and the authors conclude that the MRI appearances of papillomatosis roughly resemble those demonstrated by DCIS. On the contrary, Manganaro et al. [24] in their study about the role of breast MRI compared to galactography in patients with unilateral bloody or serous-bloody nipple discharge reported a high sensitivity of MRI in detecting papillomatosis, especially thanks to post-contrast sequences: MRI was diagnostic in all 11 cases, showing eight ductal and three regional enhancements; on pre-contrast sequences. In addition, MRI identified abnormalities in 5/11 cases, with three cystic ductal ectasia cases and two solid intraductal mass cases. Moreover, the authors found a statistically significant association between ductal enhancement and papillomatosis (*p* < 0.001), which was confirmed also at ROC analysis (Area Under the Curve [AUC] 0.790; CI 0.623–0.958), suggesting that MRI is able to differentiate between the nipple discharge causes.

### 3.2. MP and Malignancy

#### 3.2.1. MP and Malignancy in Core Needle Biopsy (CNB) and Vacuum-Assisted Biopsy (VAB)

The main problem in assessing the MP risk and the consequent management is the underestimation on the biopsy evaluation. Indeed, when benign MP diagnosis is made with CNB, the risk of upgrade to malignancy at subsequent surgical excision ranges from 3.3% [8] to 100.0% [5]. Excluding the study of Carder et al. [5] which comprises only two cases with MP, this range narrows to 3.3–10.2%.

Regarding the risk of underestimation of atypia to CNB (Table 1), the results of studies are difficult to analyze due to the different definition of atypia: MP associated to ADH [18,21] or to generally described “high-risk or atypical lesions” [8,9], or both papillomas with atypical features or papillomas with coexistent ADH [15,17,25].

The risk of underestimation after VAB was studied by Carter et al. [5] with a report of only two cases of MP: in one case, the CNB diagnosed only a fragmented papillary lesion, with the VAB suggesting papillomatosis associated to low-grade micropapillary and cribriform DCIS, which was confirmed at subsequent mastectomy; in the other case, the initial CNB suggested multiple intraductal papillomas with VAB revealing a papillary lesion associated with ADH upgraded to low-grade DCIS at subsequent open biopsy, with further DCIS and multiple papillomas found at mastectomy. Ling et al. [29] proposed fiberoptic ductoscopy-guided intraductal biopsy, but also, this technique underestimates MP: all of the MP (*n* = 12) were underestimated as solitary papilloma (*n* = 4) or ductal hyperplasia (*n* = 8) by intraductal biopsy.

#### 3.2.2. MP and Association with Premalignant/Malignant Lesions in Surrounding Parenchyma

Several studies showed the association between MP and atypia/high-risk lesions and in situ carcinomas. According to the Mayo Clinic series [4], MP diagnosed on open excisional biopsy were accompanied by a complex mixture of proliferative changes in the surrounding parenchyma, such as ductal hyperplasia (51/54, 94.4%), sclerosing adenosis (47/54, 87.0%) and radial scar (RS) (18/54, 33.3%). Similarly, Ali-Fehmi et al. [6] reported that in 72.1% of cases, atypical ductal hyperplasia (ADH) was found in tissues adjacent to MP lesions, and also, other high-risk lesions, such as lobular carcinoma in situ (LCIS), atypical lobular hyperplasia (ALH) or RS, were frequently associated.

**Figure 7:**
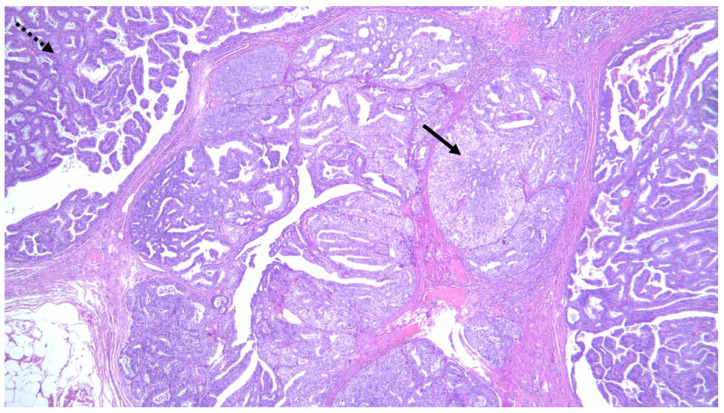
Histologic section showing multiple papillomas composed of a fibrovascular core covered with ductal epithelial and myoepithelial cells (dotted arrow) combined with foci of intraductal carcinoma (solid arrow).

On the other hand, most malignant lesions associated with a background of MP were found to be low- to intermediate-grade ductal carcinoma in situ (DCIS) [5,6,9], frequently of papillary type [20]. The presence of DCIS in this setting has been shown to have a spatial distribution; indeed, malignancy changes generally harbor within the area of MP [10,12] but also in the surrounding tissues [6] (Figure 7).

In particular, Ali-Fehmi et al. [6] retrospectively reviewed a series of 61 cases of MP retrieved from the surgical pathologic materials, including mastectomy or lumpectomy, which were divided into four categories based on initial histologic evaluation: MP without atypia (*n* = 17), MP with atypia, defined as areas within papillomas (i.e., not the accompanying tissue without papillary lesions) (AMP) (*n* = 11), MP with intraductal carcinoma (MP-DCIS) (*n* = 20) and MP with invasive carcinoma (MP-INV) (*n* = 13). The authors reported that all the MP-DCIS cases exhibited DCIS not only within or arising from the ducts involved by pre-existing papillomas but also in the surrounding tissues in 90% (18/20) of cases; similarly, 8/13 (62%) of MP-INV arose in tissues involved by MP-DCIS but 2/13 (15.4%) were present within immediately adjacent tissues.

MP tends to have wide spatial distribution, as reported by Papotti et al. [12], who analyzed a series of 18 cases with the association of MP and DCIS in surgical specimens (11 mastectomies and 7 quadrantectomies). In the mastectomy specimens, in 5/11 cases (45%), MP appeared evenly dispersed in all of the breast parenchyma, not only in the quadrant affected also by DCIS. For example, in all the quadrantectomy cases, the whole tissue was affected. Moreover, in the nine cases of MP described by Raju et al. [14], 17.4% (4/23) were bilateral. Furthermore, in cases of recurrent MP, Murad et al. [10] described that for 5/11 (45.5%) patients, it happens in the same breast after local excision, while Harjit et al. [9] observed recurrence at the same site in 3/23 (13.0%) patients, while one patient developed MP associated with DCIS in the contralateral breast after 1 year. These data highlighted the possibility of multicentric and bilateral disease.

Ohuchi et al. [11] also made a 3D reconstruction of mammary ducts on surgical specimens of patients with intraductal papillomas to analyze their growth behavior. In their study, in all patients with MP (15 cases), the lesions originated in TDLU, with some of them confined within the TDLU and others extended to the subsegmental or segmental ducts.

#### 3.2.3. MP and Risk for Breast Cancer Development

Another aspect to be addressed about the relationship between MP and malignancy is the risk of developing invasive breast carcinoma in patients affected by MP. Ali-Fehmi et al. [6] reported that 13/61 cases had invasive carcinoma, 8 of which were small (<2 cm), low grade and all cases who have known ER status were positive. Pellettiere et al. [13] reported that 4/97 patients subsequently developed biologically invasive cancer, 50% ipsilateral (2/4, developed in 1–3 years) and 50% contralateral (2/4, both developed in 4 years); similarly, in the Mayo Clinic series [4], 4/9 patients that developed breast cancers were ipsilateral to excisional biopsy location for MP, while 5/9 (55.6%) were contralateral.

These studies pointed out the necessity of risk-related measures of effect size in this subset of patients, but to date, results are conflicting. Kabat et al. [19] and Ciatto et al. [16] did not find any association between MP and a higher risk of subsequent cancer, while Lewis et al. [4] reported a significantly increased relative risk (RR) of developing carcinoma in MP without atypia (3.01 [95%CI 1.10–6.55]), which was even higher when ADH or ALH was identified within the papilloma or in the surrounding parenchyma (RR = 7.01 [95%CI 1.91–17.97]). In addition, Pellettiere et al. [13], using Kilgore’s modification of Dublin’s calculations in determining the risk of developing cancer in a 10-year period at age 45 as being 1%, reported that the risk of developing breast cancer of a woman with MP is 7.4 times greater than the expected risk in the normal population of comparable age.

### 3.3. Management

The management of MP is unclear. While most recent guidelines suggest follow-up for SP when radiologically removed with vacuum-assisted excision (VAE), no guidelines are described for papillomatosis, even if surgical excision is recommended by all the authors [9,16,31]. However, the more suitable surgical option is debated (excisional biopsy, wide local excision, mastectomy). Minimally invasive breast surgery techniques recently proposed in patients with nipple discharge, such as endoscopic papillectomy or microdiscectomy, exhibit less therapeutic efficacy in patients with multiple lesions [27,28].

To date, no study in the literature has clearly described factors to take into account to help decide the type of surgery, nor compared the prognosis of MP patients who undergo breast conservative surgery with patients treated by a mastectomy. It was recommended that if breast conservative treatment is undertaken, a clear margin of at least 10 mm should be adhered to [9]. Moreover, no guidelines reported the adequate diagnostic exams to undertake when MP is suspected/diagnosed or after its treatment (if conservative surgery is chosen).

## 4. Discussion

Classic papillomatosis, also referred to as peripheral/multiple papillomas, is characterized by papillary proliferations within multiple terminal duct-lobular units or in the distal branches (terminal ducts) of the duct system [1,3]. It must be differentiated from other papillary lesions of the breast such as juvenile papillomatosis, which is a rare benign proliferative lesion occurring in young women and histologically characterized by a constellation of proliferative changes and large cysts [32], or from other proliferative changes such as infiltrating epitheliosis, which refers to a complex sclerosing lesion composed of radiating ducts and lobules in a scleroelastotic stroma [33].

As for imaging presentation, there is only little evidence about MP features on different imaging modalities, with a wide spectrum of appearances. Mammography is limited, with poor sensitivity and specificity [14,22,23]; breast ultrasound can be helpful to detect papillary lesions, but tissue sampling is always necessary to confirm the ultrasound suspect [23,26]. MRI is useful for determining the extent of the disease and to identify the connections with the ductal system. The MRI protocol should include acquisitions before intravenous contrast administration, to show dilated ducts in which papillomas can be detected as filling defects, and after intravenous contrast administration, due to the significant enhancement of papillary lesions [24,26].

The accurate diagnosis of papillary lesions is a challenge also for pathologists, and the underlying causes of this diagnostic difficulty are multiple. First of all, there are difficulties in pathologic interpretation on biopsy samples, particularly in the distinctions between benign papillomas, papilloma with ADH/DCIS and papillary carcinomas. These problems mainly arise in overlapping morphological features and, sometimes, immunohistochemistry staining could be not helpful [34]. Second, there may be a risk of underestimation of malignancy or atypia due to sampling error. When a solitary papilloma is diagnosed, the Second International Consensus Conference on lesions of uncertain malignant potential in the breast (B3 lesions) reported that therapeutic excision with VAB or VAE is recommended to avoid potential sampling error and permitting to avoid surgical excision and justify the surveillance option [31]. In the case of papillomatosis, this option cannot obviously be invoked. Moreover, conversely to solitary papillomas, in MP, the risk of underestimation is not only connected to sampling errors of the lesions but also of the surrounding parenchyma. Indeed, MP were reported to be frequently associated with malignant or atypical/high-risk lesions (such as ADH) in the breast tissue surrounding the area of papillomatosis [4,6].

Moreover, as reported above, MP tends to have wide spatial distribution, with all breast parenchyma involved and frequent bilateral lesions [12,13,14]. This aspect sets out two considerations. First, when papillomatosis diagnosis is made, a careful investigation of multifocality to define the real extent of the pathology is mandatory. To date, no evidence is available about whether mammography and ultrasound alone are sufficient to define the real involvement of the breast or the presence of contralateral lesions or if MRI, which is demonstrated to have a higher sensitivity, could be more suitable for this intent [24,25]. The not accurate evaluation of actual size of the lesion, which may be larger than that appreciated by clinical and imaging findings, or the presence of satellite lesions, may give rise to the potential risk of incomplete excision and recurrence. Indeed, it is not clear if the higher risk of developing breast cancer in women affected by papillomatosis is linked to the fact that MP can be considered indicators of high-risk subjects or if it is a precancerous lesion that may slowly progress into cancer when it is not properly and completely removed because of limited surgery or inadequate pre-surgical evaluation of the extension, multifocality or bilaterality of the disease [10,12,14]. Moreover, the frequent association of papillomatosis with other high-risk lesions, especially ADH, is another confounding factor which may be responsible for the higher risk of these women to develop breast cancer in the homolateral and in the contralateral breast [4,6].

However, if on one hand, all authors believe that surgical excision is unavoidable when a diagnosis of papillomatosis is made; on the other hand, there is still a lack of evidence about the correct surgical treatment (excisional biopsy, wider surgical excision, mastectomy) [9,13]. A careful evaluation of symptoms (nipple discharge, palpable mass) and patient’s history (e.g., previous breast cancer or histologic atypical hyperplasia) is mandatory to tailor the management and to reduce overtreatment and spare patients from unnecessary anxiety or high healthcare costs. Moreover, the patient’s choice in decision making is paramount in such cases. As part of the informed consent process, patients must receive sufficient information also about the possibility of underestimation and recurrence of the disease. Finally, a careful pathology/radiology correlation and a multidisciplinary approach is advocated for these patients [31].

There is also a lack of evidence about the time of follow-up and the imaging method to be used to ensure early detection of recurrence or subsequent breast cancer in treated patients. Further studies are needed to enable a more personalized approach; new diagnostic tools such as artificial intelligence could find their place in tailoring the diagnostic process and determining the appropriate imaging follow-up.

Despite the great interest and existing evidence about the link between papillomatosis and breast cancer, a number of limitations remain. First, the rarity of this pathology and the inconsistent terminology (with different definitions of papillomatosis), as shown in Table 1, make the review of current evidence very difficult. Second, an extreme heterogeneity in the pre-surgical evaluation of the extension of this disease and about surgical choice could be, at least in part, responsible for the conflicting results of the published articles about the risk of developing breast cancer.

## 5. Conclusions

Papillomatosis can be considered a challenge for all breast specialists. Its appearance varies clinically, radiologically and pathologically, so familiarity with its features is required to make the correct diagnosis. Its association with malignancy is still debated, and clinical and imaging factors associated with increased breast cancer risk need to be further investigated. In the era of personalized medicine, further studies are needed to evaluate the factors to take into account to plan management, the time of follow-up and the imaging method to be used.

## Figures and Tables

**Table 1:** Overview of the included studies.

Study			Subjects		Clinical Findings	Imaging Findings	Upgrade Rate *	Follow-Up Time	Outcome	Additional Findings
Reference	Design	Definition of MP	Number	Age (Years)						
Carder 2008 [5]	Retrospective cohort, patients with B3/B4 lesions at CNB, with the words ‘‘papillary’’ or ‘‘papilloma’’ in the final diagnosis, followed by SE or mammotome excision	NR	2	NR	48/61 Palpable breast mass (78%); 10/61 Nipple discharge (16%)	**MX**: 2/2 segmental indeterminate calcifications **US**: 1 negative; 1 altered echogenicity with nodules	- CNB vs. VAB: 2/2 (100%): 1 MP + ADH; 1 MP + DCIS - SE vs. CNB: 2/2 (100%) - SE vs. VAB: 1/2 (50%, from MP + ADH to DCIS)			
Ali-Fehmi 2003 [6]	Retrospective cohort, patients with MP in all available pathologic materials (mastectomy/lumpectomy)	≥5 papillomas in at least 2 non-consecutive tissue blocks	MP without atypia (n 17) MP with atypia (n 11) MP with DCIS (n 20) MP with invasive BC (n 13).	36–84 (mean, 57.8)	NR	NR	NR	MP without atypia: 2–10 years; mean, 47.2 months MP with atypia: 7 months-6 years (mean, 61 months) MP with DCIS: 4–125 months; mean, 40.5 months MP with invasive BC: 16–110 months (mean, 59 months)	MP without atypia: no R or BC MP with atypia: One ipsilateral mucinous carcinoma (2 years after diagnosis of MP) MP with DCIS: 3 contralateral BC (2 IC and 1 DCIS, 11–36 months after diagnosis of MP-DCIS) MP with invasive BC: 1 died from disease, 2 contralateral disease (DCIS 8 years after diagnosis, 1 invasive lobular BC 5 months after diagnosis)	- In 4/17 cases of MP without atypia, ADH in the adjacent tissues - All of the cases exhibited DCIS within or arising from ducts involved by pre-existing papillomas but also in the surrounding tissues - 62% of MP with invasive BC cases arose in tissues involved by MP-DCIS and 2 within immediately adjacent tissues.
Carter 1977 [7]	Retrospective cohort, patients with MP in all available pathologic materials	Multiple discrete papillomas >3 mm without significant accompanying diffuse hyperplasia or papillomatosis	6	NR	NR	NR	NR	at least 5 years	2/6 (33%) carcinoma (type not specified)	
Han 2018 [8]	Retrospective cohort, patients with IDPs without atypia at CNB (14G needle)	≥2 lesions separated by normal breast tissue in imaging eventually proven to be benign MP on pathologic examination	91	NR	NR	NR	3/91 (3.3%) upgraded to malignancy (all DCIS); 4/91 (4.4%) upgraded to high risk lesions (ADH/LIN)	NR	NR	
Harjit 2006 [9]	Retrospective cohort, patients with MP in all available pathologic materials (FNA/CNB/SE/mastectomy)	≥5 papillomas in the same quadrant or in at least two consecutive surgical pathology tissue blocks	23 total: 13 CNB (10 without atypia, 1 with ADH and 2 with DCIS); 9 excisional biopsy; 1 mastectomy	NR	18 screen-detected lesions (non-palpable); 5 palpable lumps	NR	- MP without atypia: 2/10 (20.0%) upgraded to MP + DCIS and 1/10 (10.0%) upgraded to MP + ADH - MP + ADH: 1/1 (100%) upgraded to MP + DCIS - MP + DCIS: no upgrade	4.1 years (range 1–10 years)	3/23 recurrence of MP at the same site and 1/23 MP + DCIS in the contralateral breast after 1 year.	
Lewis 2006 [4]	Retrospective cohort, patients with IDPs on open excisional biopsy	≥5 papillomas in 2 non-consecutive tissue blocks	54: 41 without atypia and 13 with atypia (ADH/ALH within papilloma or surrounding parenchyma)	NR	NR	NR	NR	16 years	RR of developing carcinoma: MP without atypia 3.01 (95%CI 1.10–6.55); MP with atypia 7.01 (95%CI 1.91–17.97).	- In 93% of cases, MP were accompanied by a complex mixture of proliferative changes (sclerosing adenosis, usual ductal hyperplasia and RS) - 5/9 cancers contralateral to MP
Murad 1981 [10]	Retrospective cohort, patients with IDPs in surgical specimens	A lesion that involves many adjacent lactiferous ducts by a papillary process	21	NR	NR	NR	6/21 (28.6%) showed malignancy changes within the area of MP (3/6 invasive)	NR	50% recurrence rate after local excision	
Ohuchi 1984 [11]	Retrospective cohort, patients with MP in surgical specimens	NR	15	NR	NR	NR	5/15 (33.3%) showed malignancy changes within the area of MP (all DCIS)	NR	NR	100% of MP involved TDLUs (some confined within the TDLU and others extended to the subsegmental/segmental level)
Papotti 1984 [12]	Retrospective cohort, patients with MP + DCIS in surgical specimens	From a minimum of 5 to a maximum of 153 papillomas	18	mean 51.3	44.4 % nipple discharge	NR	NR	17 months	1/7 patients who underwent quadrantectomy recurred four years later (mastectomy was then performed)	Spatial distribution of papillomas and DCIS: MP only in the quadrant affected also by carcinoma in 6/11 mastectomy specimens; whole quadrant affected in 7/7 quadrantectomy cases
Pellettiere 1970 [13]	Retrospective cohort, patients with MP in surgical specimens	NR	97	mean 45.5 (range 18–71 years)	- 28/97 (28.9%) nipple discharge (11/28 bloody) - 77/97 palpable mass vs. 20/97 vague thickenings	NR	NR	5–18 years	4/97 subsequently developed biologically invasive cancer: 2/4 ipsilateral developed in 1–3 years, while the 2/4 contralateral both developed in 4 years	- 17/97 bilateral disease - The risk of a woman with MP is 7.4 times greater than the expected risk in the normal population of comparable age (Kilgore’s modification of Dublin’s calculations)
Raju 1996 [14]	Retrospective cohort, patients with MP on open excisional biopsy	NR	10 MP with ADH and 13 MP without atypia	NR	MP with ADH: 3/10 nipple discharge, 1/10 palpable mass (N.R. for MP without atypia)	**MX**: MP with ADH: 2/10 asymmetric density, 5/10 masses (NR for MP without atypia)	NR	NR	MP + ADH: 1/7 ipsilateral DCIS (intermediate to high-grade), 2 contralateral IC MP without atypia: 1/13 contralateral invasive BC	In 4/23 cases, MP was bilateral
Chang 2011 [15]	Prospective study, patients with SE of non-malignant papillary lesions diagnosed at US-guided 11-gauge VAB	NR	7	NR	NR	NR	2/7 (28.6%) upgraded to MP + ADH	NR	NR	
Ciatto 1991 [16]	Retrospective cohort, patients with IDPs on surgical specimens (complete resection or mastectomy)	NR	84	NR	All patients self-referred for nipple discharge	NR	NR	2 to 14 years (average, 6.62 years)	RR of developing carcinoma 1.40 (95%CI 0.04–7.79)	
Fu 2012 [17]	Retrospective cohort, CNB-diagnosed papillary lesions of the breast with subsequent excisional biopsy	NR	109: 77 without atypia, 25 with atypia	NR	NR	NR	- 11/109 (10.1%) upgraded to malignant lesions - MP without atipia: 20/77 (26.0%) upgraded to atypical /malignant - MP with atypia: 7/25 (28.0%) upgraded to malignant	NR	NR	
Gendler 2004 [18]	Retrospective cohort, biopsy-diagnosed papillary lesions of the breast with subsequent SE	≥5 papillomas in at least 2 consecutive surgical pathology tissue blocks	11	NR	NR	NR	5/11 (45%) upgraded to breast cancer and 3/11 (27%) upgraded to MP + ADH	NR	NR	
Kabat 2010 [19]	Nested case-control study (Cases: women with biopsy for benign breast disease including IDP and who subsequently developed BC; controls: individually matched to cases women with biopsy for benign breast disease who did not develop breast cancer in the same FUP interval as that for the cases)	≥3 papillomas	11	NR	NR	NR	NR	15.4 years	Unadjusted OR 1.38 (95%CI 0.56–3.44) Adjusted OR 1.36 (95%CI 0.52–3.51)	
Koo 2013 [20]	Retrospective cohort, biopsy-diagnosed papillary lesions of the breast with subsequent SE	NR	98	NR	NR	NR	10/98 (10.2%) papillary DCIS	NR	NR	Use of IHC may decrease upgrade-to-malignancy rate for benign papillary lesions on US-guided 14G CNB
Liberman 2006 [21]	Retrospective cohort, biopsy-diagnosed papillary lesions of the breast with subsequent SE or >2 years FUP	NR	10: 7 surgically excised and 3 stable at FUP	NR	NR	NR	2/10 (20.0%) upgraded to breast cancer and 3/10 (30.0%) upgraded to MP + ADH	NR	NR	In 4/7 surgically excised MP, other high-risk lesions were founded (3 ADH, 1 RS)
Sohn 2013 [22]	Retrospective cohort, 14G CNB-diagnosed papillary lesions of the breast with subsequent VAB or SE	NR	17	NR	NR	NR	2/17 (11.8%) upgraded to atypical papillomas or papillomas with ADH	NR	NR	
Cardenosa 1991 [23]	Retrospective cohort, biopsy-diagnosed papillary lesions of the breast	NR	14 peripheral MP and 12 central MP	- peripheral MP: 52 (38–72) - central MP: 52 (30–77)	- peripheral MP: 11/14 asymptomatic; 2/14 palpable abnormalities; 1 bilateral clear nipple discharge - central MP: 12/12 nipple discharge (5 bloody, 7 serous/clear)	**MX**: - peripheral MP: peripheral calcifications (5/14), clusters of nodules (2/14), masses (1/14), spiculated opacities (1/14), asymmetric opacities (1/14), asymmetric tissues with calcifications (1/14), lobulated solid mass at **US** (1/14), no imaging finding in symptomatic patient (1/14) - central MP: 11/12 normal mammography, 1/12 asymmetric prominent ducts	NR	NR	NR	The tissue adjacent to peripheral MP contained apocrine metaplasia, sclerosing adenosis, FEA, ADH, LCIS, RS
Manganaro 2015 [24]	Retrospective cohort, unilateral discharge patients who performed galactography and MRI	NR	11	NR	NR	**MRI**: - Pre-contrast: 3 cystic ductal ectasia cases and 2 solid intraductal mass - Post-contrast: 8 ductal and 3 regional enhancements	NR	NR	NR	- Galactography identified the pathology in 5/11 cases (55% false negative cases). - Statistically significant association between ductal enhancement and papillomatosis (*p* < 0.001).
Son 2009 [25]	Retrospective cohort, patients who underwent surgery due to papillomas of the breast and performed 3D fast low-angle shot (FLASH) dynamic breast MRI	NR	3	41.7 ± 12.9 (27–51)	2/3 palpable mass, 1/3 bloody nipple discharge	**MX**: 2/3 microcalcifications **US**: 3/3 multiple masses **MRI**: 1/3 multiple nodular enhancement; 1 ductal non-mass enhancement, 1 segmental non-mass enhancement	NR	NR	NR	
Sarica 2018 [26]	Retrospective cohort, patients with a pathologic diagnosis of papillary lesion who performed MRI and US	NR	11	41.45 ± 7.7	1/11 palpable mass, 3/11 unilateral nipple discharge	**US**: 3/11 dilated duct partially/completely filled with intraluminal content; 1/11 mass with ductal relation or intracystic mass; 6/11 heterogeneous tubular nonmass-like hypoechoic area or mass related to multiple dilated ducts; 1/11 occult **MRI**: 3/11 dilated duct and intraductal focal mass on T2; 2/11 Dilated duct and pre-contrast high T1 signal; 2/11 mass with crescentic peripheral fluid; 3/11 mass related with dilated duct-ductal contrast enhancement; 1/11 linear-ductal contrast enhancement; 3/11 segmental contrast enhancement	NR	NR	NR	
Bender 2009 [27]	Retrospective cohort, patients who underwent ductoscopy for pathologic nipple discharge	NR	5	NR	Nipple discharge	NR	NR	NR	NR	After endoscopic papillomectomy, nipple discharge stopped in all patients without recurrences
Kamali 2014 [28]	Prospective cohort, patients who underwent ductoscopy for pathologic nipple discharge and diagnosed with MP on final histology	NR	14	NR	Nipple discharge	**MX**: 4/14 soft tissue mass; 1/14 microcalcifications; 2/14 distortions	NR	NR	NR	Ductoscopy was diagnostic in 8/14 patients
Ling 2009 [29]	Retrospective cohort, patients who underwent ductoscopy for pathologic nipple discharge and subsequent MP diagnosis on final histology	NR	12	NR	Nipple discharge	NR	NR	NR	NR	All MP were underestimated as solitary papilloma (4/12), or ductal hyperplasia (8/12) by intraductal biopsy.
Liu 2015 [30]	Prospective cohort, patients who underwent ductoscopy for pathologic nipple discharge and diagnosed with MP on final histology	NR	42	NR	Nipple discharge	NR	NR	NR	NR	Ductoscopy was diagnostic in 24/42 patients

Abbreviations: ADH, atypical ductal hyperplasia; ALH, atypical lobular hyperplasia; BC, breast cancer; 95%CI, 95% confidence interval; CNB, core needle biopsy; DCIS, ductal carcinoma in situ; FEA, flat epithelial atypia; FNA, fine needle aspiration; FUP, follow-up; IDP, intraductal papilloma; LCIS, lobular carcinoma in situ; LIN, lobular intraepithelial neoplasia; MP, multiple papillomas; MRI, magnetic resonance imaging; MX, mammography; NR, not reported; OR, odds ratio; R, recurrence; RR, relative risk; RS, radial scar; SE, surgical excision; SD, standard deviation; TDLU, terminal duct lobular unit; US, ultrasound; VAB, vacuum assisted biopsy. * Upgrade rate: was defined as the proportion of lesions initially diagnosed as benign papillomas and found atypical or classified as DCIS or invasive cancer after VAB or surgical excision.

## Data Availability

The data presented in this study are available on request from the corresponding author.
